# Hygiene and Public Health

**Published:** 1891-03

**Authors:** 


*Hygiene and Public Health.* By B. ARTHUR WHITELEGGE, M.D., B.Sc. Lond., D.P.H. Camb. London: CASSELL & Co. 1890.

*Text-Book of Hygiene.* By GEORGE H. ROHÉ, M.D. Second Edition. Philadelphia: F. A. DAVIS. 1890.

*Aids to Sanitary Science.* By FRANCIS J. ALLAN, M.D. London: BAILLIÈRE, TINDALL & COX. 1890.

The student of public health who carefully reads Dr. Whitelegge's manual will have gained a very concise and practical insight into the principles underlying the modern practice of hygiene, and will have laid a very useful foundation for subsequent study. Particularly well written are the chapters on infection, disinfection, and the specific diseases; and here, as also in those dealing with notification, schools and infection, the duties of medical officers of health, and the framing of by-laws, there is evidence that the book is the outcome of actual and valuable experience. The chapters on sanitary law and statistics are necessarily brief; but here again the text may be safely accepted as a trustworthy introduction to the larger treatises. It would be difficult to recommend a better guide to inexperienced health officers.

Dr. Rohé's work forms a very handsome volume, and is pleasant reading; but it is hardly convincing. The chapter dealing with the analysis of water, for example, is far too general and incomplete to be taken as a sufficient guide by the student without consulting the supplementary text-books indicated at the end of the chapter, and yet the book is intended to be a "comprehensive treatise." The history of epidemic diseases in Chapter XIX. *et seq.* is, however, very interesting; and the chapter on antiseptics, disinfectants, and deodorants is fairly complete and trustworthy. Taken as a whole, the work contains a considerable amount of varied information of considerable interest, somewhat popularly treated.

Dr. Allan's *Aids to Sanitary Science* is much better than many of its class. The information it contains appears to have been carefully selected, and to be fairly up to date. Used as a lecture note-book in order to refresh the memory, it may be found useful; but it is of course not intended for, and its necessarily brief style would render it most exasperating if used as, a text-book.

